# Editorial: Women in veterinary regenerative medicine: 2021

**DOI:** 10.3389/fvets.2022.1085747

**Published:** 2022-11-29

**Authors:** Cristina L. Esteves

**Affiliations:** The Roslin Institute and Royal (Dick) School of Veterinary Studies, The University of Edinburgh, Edinburgh, United Kingdom

**Keywords:** Veterinary Regenerative Medicine, women in science, mesenchymal stem/stromal cells, Science Technology Engineering Mathematics (STEM), gene therapy, repair

Women representation in STEM has increased over the years, although gender unbalance is still notorious with only around 30% of the workforce represented by women (1). Surprisingly, this does not apply to the veterinary profession in the western world (2), although women still remain largely underrepresented in senior leadership positions, on veterinary practice and other occupations, including Editorial positions in veterinary science journals (2–4).

This Research Topic aims to illustrate recent progress in Veterinary Regenerative Medicine research and highlights the significant contribution of female scientists in advancing this field.

Veterinary Regenerative Medicine has seen a great expansion in the last two decades, driven by intense research toward the design and clinic application of new therapies to enhance repair of tissues damaged by trauma or disease in animals. This has been largely driven by the identification of cells and other biological products with healing potential, with adult mesenchymal stem/stromal cells (MSCs) being a main player in the field. Through multidisciplinary approaches, the use of cells or other biologicals, alone or in association with scaffolds, has produced a diverse number of products and applications targeting a variety of tissues and diseases in domestic animals, with relevance for human health in some instances.

Despite considerable advances to date, significant improvements are still needed on standardization, scale-up production and enhancement of the properties of cells and other therapeutic products, alongside with optimization of delivery methods and efficacy testing, as well as better understanding of host response to interventions. Such improvements will, in the future, benefit animal health and contribute to the approval of new medical products by relevant health agencies, ultimately facilitating their commercialization.

The articles published in this Research Topic report on and shed new light into some of the above-indicated issues in Veterinary Regenerative Medicine. A common theme is the study or clinical testing of cells, MSCs and other, with articles covering isolation, characterization. and improvement of MSC culture (Harman et al.; Heyman et al.; Hagen et al.; Even et al.), as well as enhancement of repair therapies using genetic modification (Thampi et al.).

Studies on MSCs have so far focused largely on mammalian species. In this Research Topic, expanding from previous work in equine peripheral blood-derived MSCs (PB-MSCs), the van de Walle group reports on the isolation and characterization of chicken PB-MSCs. These cells displayed typical MSC features including tri-lineage differentiation and surface markers, as well as *in vitro* antimicrobial and tissue repair properties. Their results provide the basis for further study on the biology of avian MSCs and the development of cell-based therapies for chicken, an area of significant potential for the poultry industry.

Two of the articles in this Research Topic, one using equine adipose tissue- (Hagen et al.) and the other bone marrow-derived MSCs (Even et al.), explore equine platelet lysate and serum as substitutes for FBS in cell culture medium, showing positive and encouraging results on functional and immunomodulatory properties. The use of xenogenic-free medium is desirable to avoid immune responses in MSC therapies, however, standardization of methods of production will be essential to ensure consistency when using these supplements. Cost, scale-up production and allogeneic immunogenicity are other aspects to be considered when using alternative media supplements. Switching to commercial FBS-free culture media will be key for veterinary MSC and other cellular therapy applications, and more research on media composition will be needed in the future to respond to these needs.

Another paper in this series (Heyman et al.) describes the development of a method to quantify MSC tri-lineage differentiation using the NIH free-of-charge programme, Image J. The analysis is based on color deconvolution of cell differentiation images after using classical chemical staining protocols. This method can be used to evaluate MSC differentiation capacity quantitatively, contributing to the characterization and optimization of these cell preparations for therapeutic applications or basic studies.

Osteoarthritis, a degenerative joint disease that can evolve into painful and debilitating stages, affects both humans and companion animals. Currently used treatments are intended to slow down degeneration, promote repair or ameliorate inflammation, commonly targeting the disease indirectly, with variable outcomes and side effects. New developments in genetic therapy have opened up the prospect of improved treatments for osteoarthritis with prolonged, site-specific, long-lasting effects. In that regard, Thampi et al. gives an overview on gene therapy, focusing on the horse both as a patient and a translational model. The review covers *ex vivo* gene therapy and *in vivo* approaches, using viral and non-viral vectors, and gives examples of preclinical studies, including those performed by the authors themselves. Aspects hindering the advance of these therapies are critically discussed, highlighting the advances and constraints hampering the development of effective and affordable therapies both for humans and the horse.

The articles published in this Research Topic highlight the quality of science produced by women, crucially contributing to the advance of research on relevant aspects of Regenerative Veterinary Medicine and One health.

## Author contributions

CE wrote the manuscript and approved the submitted version.

## Conflict of interest

The author declares that the research was conducted in the absence of any commercial or financial relationships that could be construed as a potential conflict of interest.

## Publisher's note

All claims expressed in this article are solely those of the authors and do not necessarily represent those of their affiliated organizations, or those of the publisher, the editors and the reviewers. Any product that may be evaluated in this article, or claim that may be made by its manufacturer, is not guaranteed or endorsed by the publisher.
